# Supplementary material for: Atomic-scale probing of short-range order and its impact on electrochemical properties in cation-disordered oxide cathodes
Source: Nat Commun. 2023 Nov 17;14:7448. doi: 10.1038/s41467-023-43356-2 (PMC10656575; doi:10.1038/s41467-023-43356-2)
Supplement: Supplementary file 1 — Supplementary Information [file 41467_2023_43356_MOESM1_ESM.pdf]

Supplementary Information

Atomic-scale probing of short-range order and its impact on electrochemical properties in cation-disordered oxide cathodes

Linze Li[1]†, Bin Ouyang[2,3]†*, Zhengyan Lun[2,4], Haoyan Huo[2], Dongchang Chen[5], Yuan Yue[5], Colin Ophus[6], Wei Tong[5], Guoying Chen[5], Gerbrand Ceder[2]* and Chongmin Wang[1]*

[1]Environmental Molecular Sciences Laboratory, Pacific Northwest National Laboratory, 902 Battelle Boulevard, Richland, WA 99354, USA

[2]Department of Materials Science and Engineering, University of California – Berkeley, 328 Hearst Mining Memorial Building, Berkeley, CA 94720, USA

[3]Department of Chemistry and Biochemistry, Florida State University, Tallahassee, FL 32306, USA

[4]School of Chemical Sciences, University of Chinese Academy of Sciences, Beijing 101408, China

[5]Energy Storage and Distributed Resources Division, Lawrence Berkeley National Laboratory, Berkeley, CA 94720 USA

[6]National Center for Electron Microscopy, Molecular Foundry, Lawrence Berkeley National Laboratory, Berkeley, CA 94720, USA.

*Correspondence to: bouyang@fsu.edu, gceder@berkeley.edu, chongmin.wang@pnnl.gov

†These authors contributed equally

Supplementary Figures

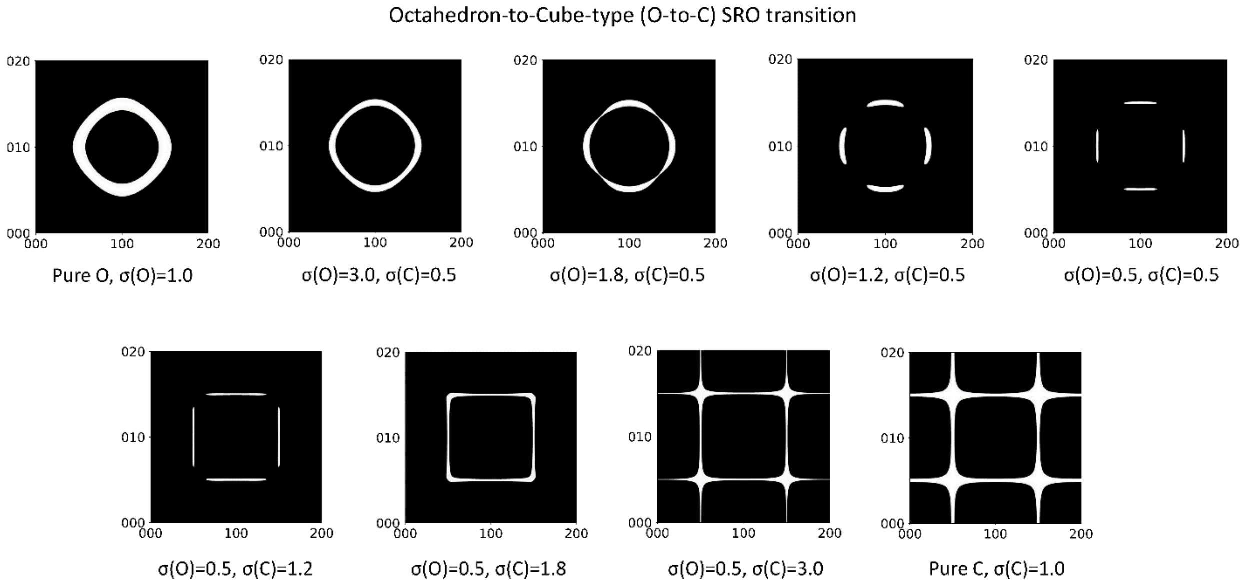

Figure S1. Calculated diffraction locus for the octahedron-type-to-cube-type SRO transition.

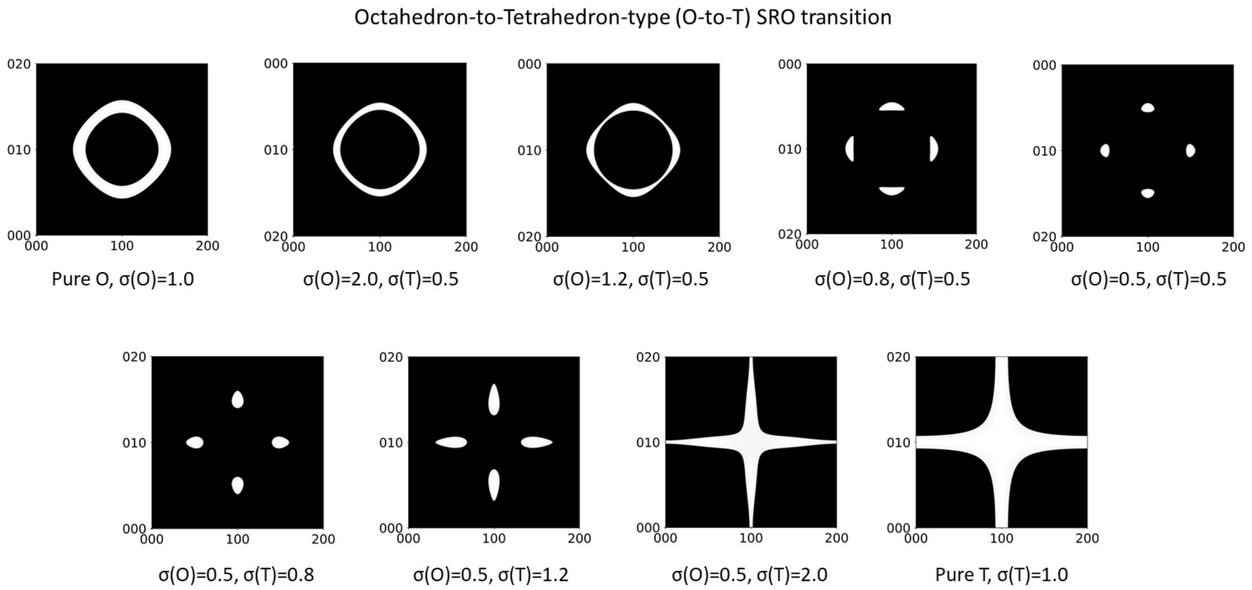

Figure S2. Calculated diffraction locus for the octahedron-type-to-tetrahedron-type SRO transition.

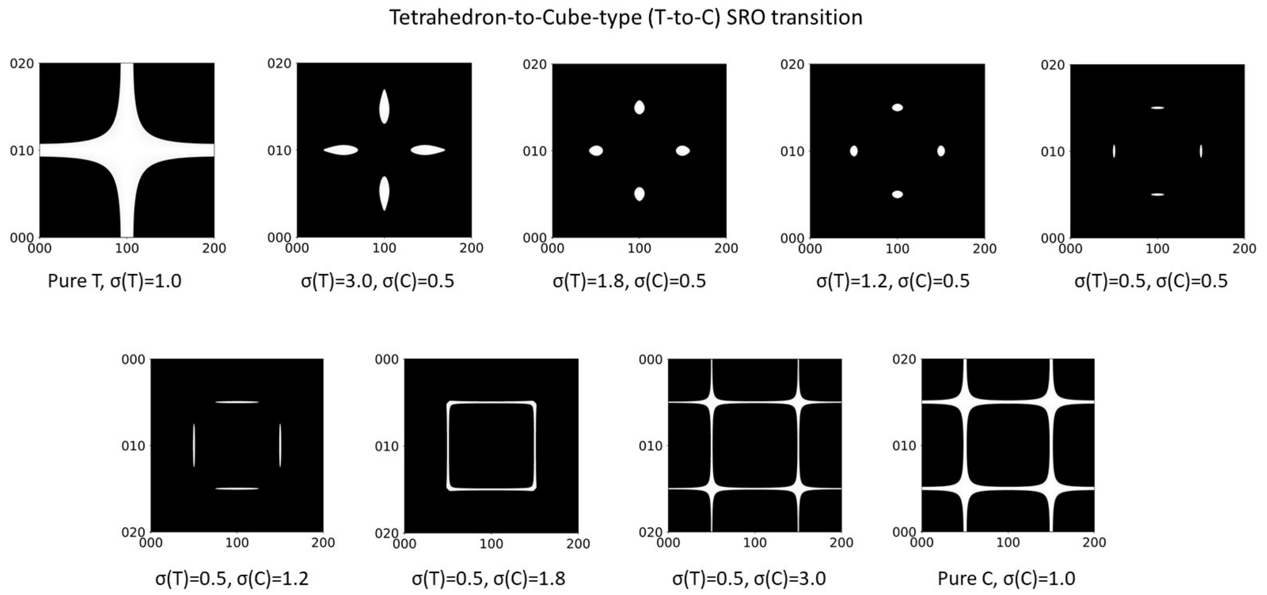

Figure S3. Calculated diffraction locus for the tetrahedron-type-to-cube-type SRO transition.

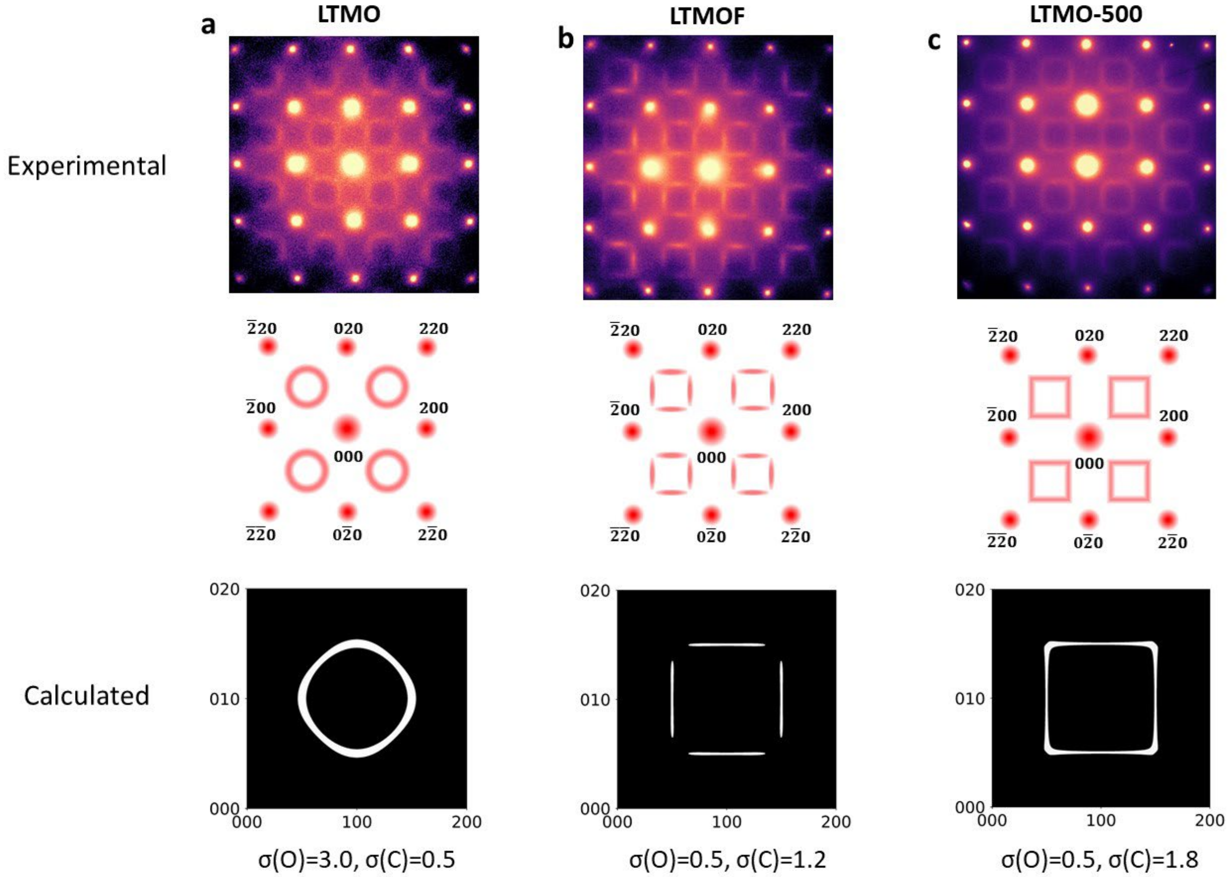

Figure S4. Comparison between experimental electron diffraction patterns and calculated diffraction locus for three materials systems. (a) LTMO; (b) LTMOF; (c) LTMO-500.

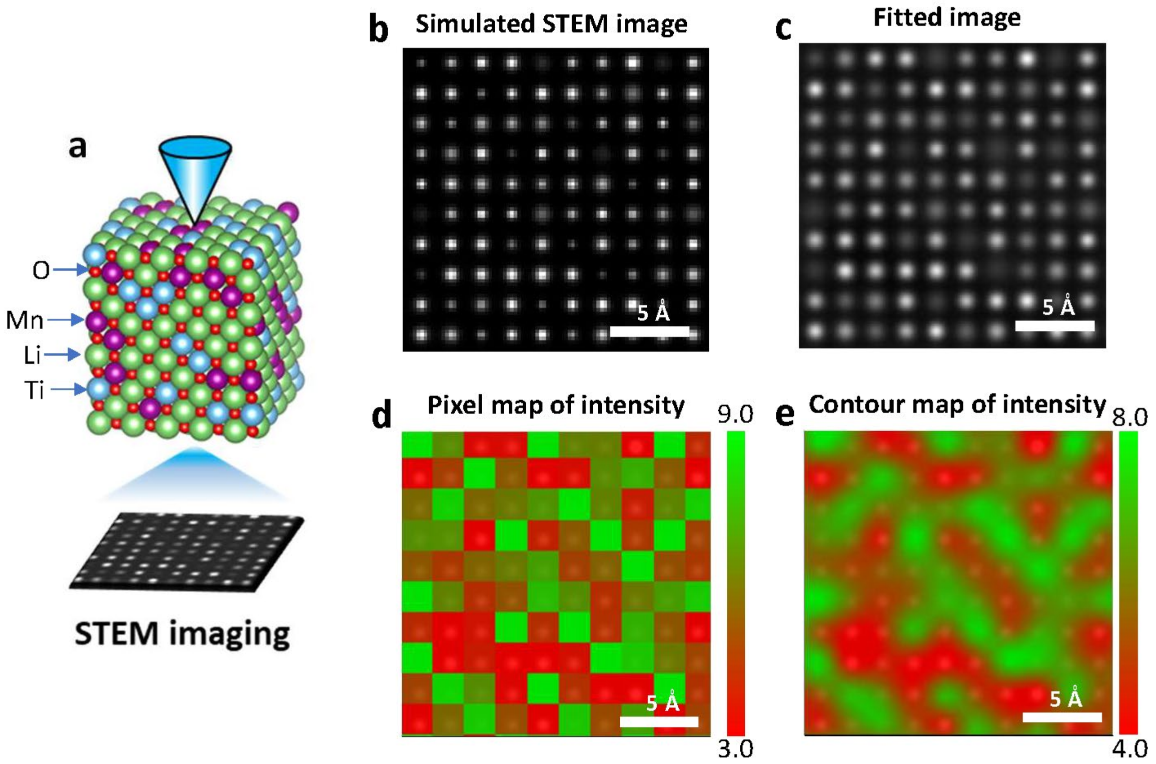

Figure S5. STEM image and Pixel map of intensity. (a) A STEM *Z*-contrast image is simulated from the 3D supercell structure calculated by the Monte Carlo simulations (red: O, . Purple atom is Mn3+, light blue atom is Ti4+, and the green atom is Li+. (b) The simulated STEM *Z*-contrast image. (c) Replot of the fitted *Z*-contrast image, after the image was processed by fitting each bright-contrast dot representing the atomic column with a two-dimensional Gaussian function. (d-e) Pixelated (d) and contour (e) color maps of intensity distributions for the atomic columns in the *Z*-contrast image. In the intensity maps, the green and red colors correspond to lower and higher intensities, respectively.

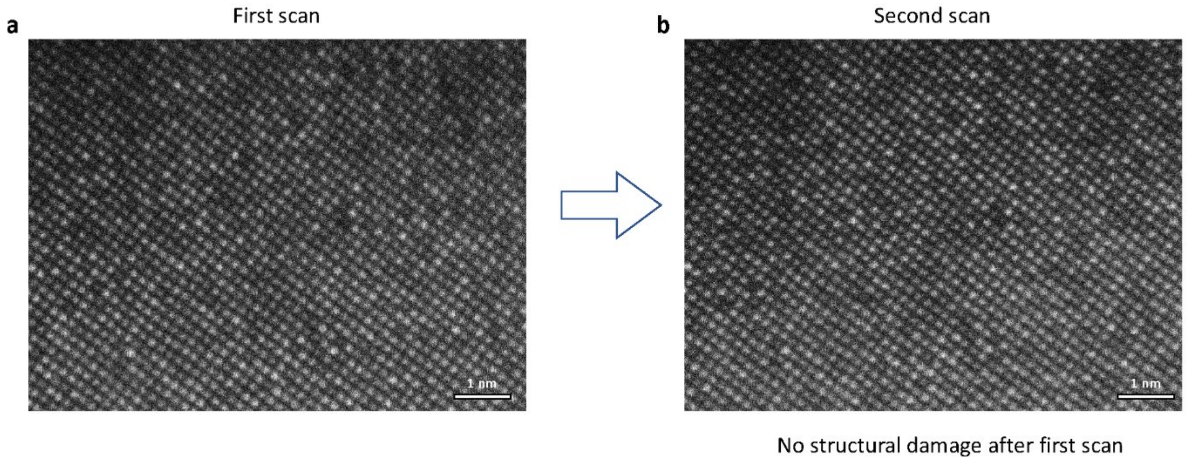

Figure S6. Evaluation of electron beam effect. Two consecutively captured STEM *Z*-contrast images of the same local region in LTMOF. (a) After capturing the first image, (b) there is no structural damage induced by the electron beam.

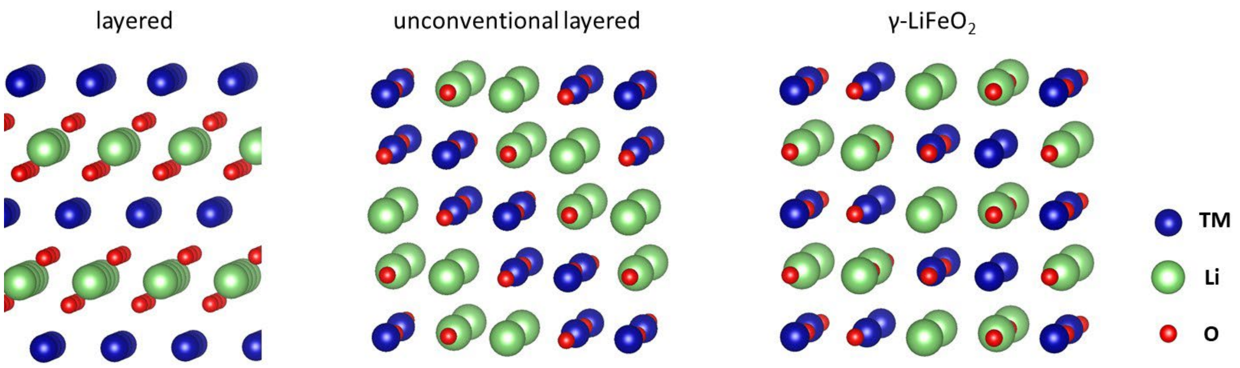

Figure S7. Atomic models of long-range ordered layered, unconventional layered, and γ-LiFeO$_2$ structures. TM represents transition metal.

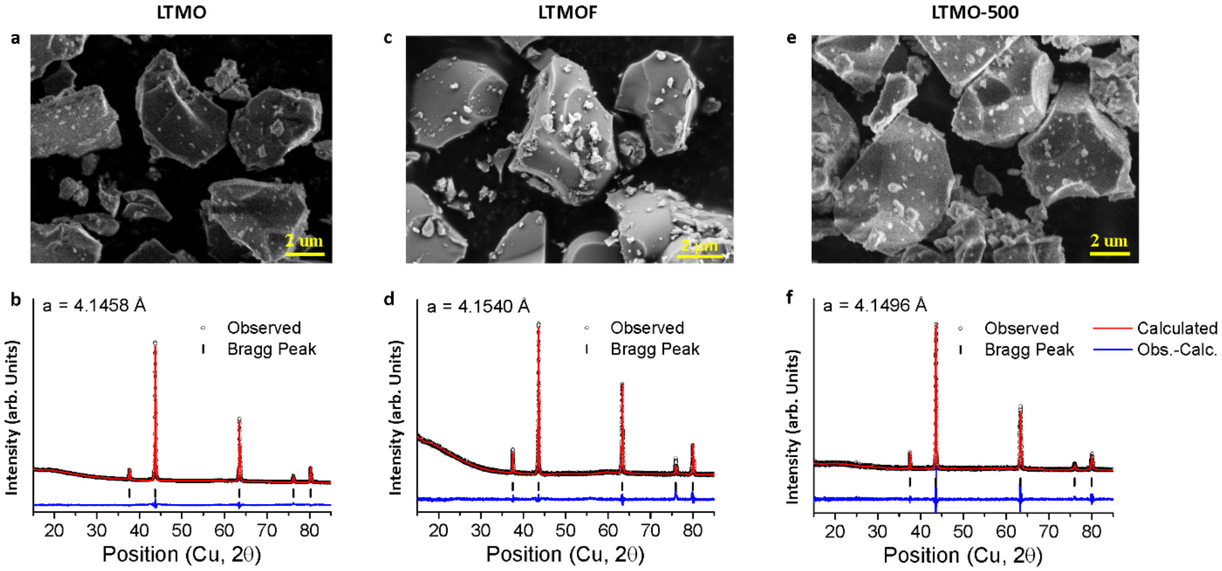

Figure S8. Characteristics of the materials. SEM images and XRD patterns of LTMO (a-b), LTMOF (c-d), and LTMO-500 (e-f).

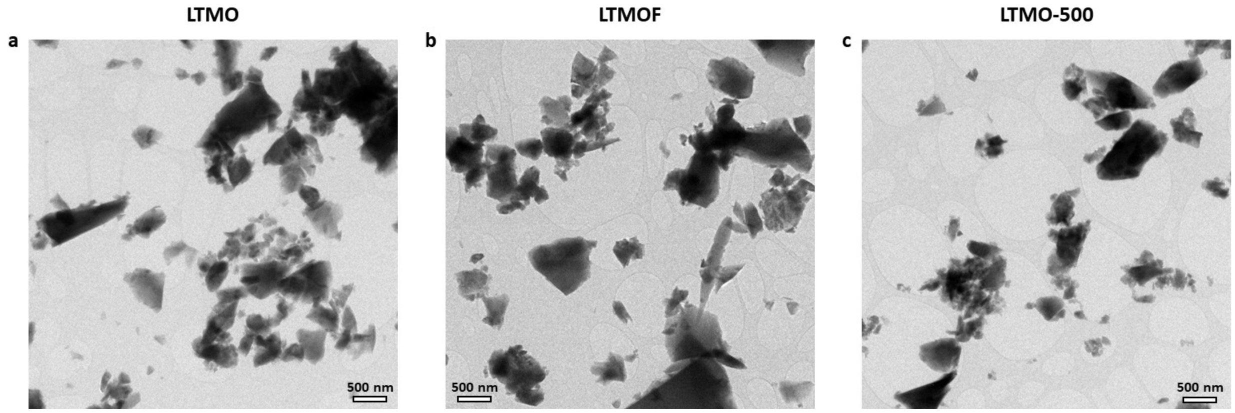

Figure S9. Morphological features of cathodes. TEM images of LTMO (a), LTMOF (b), and LTMO-500 (c).

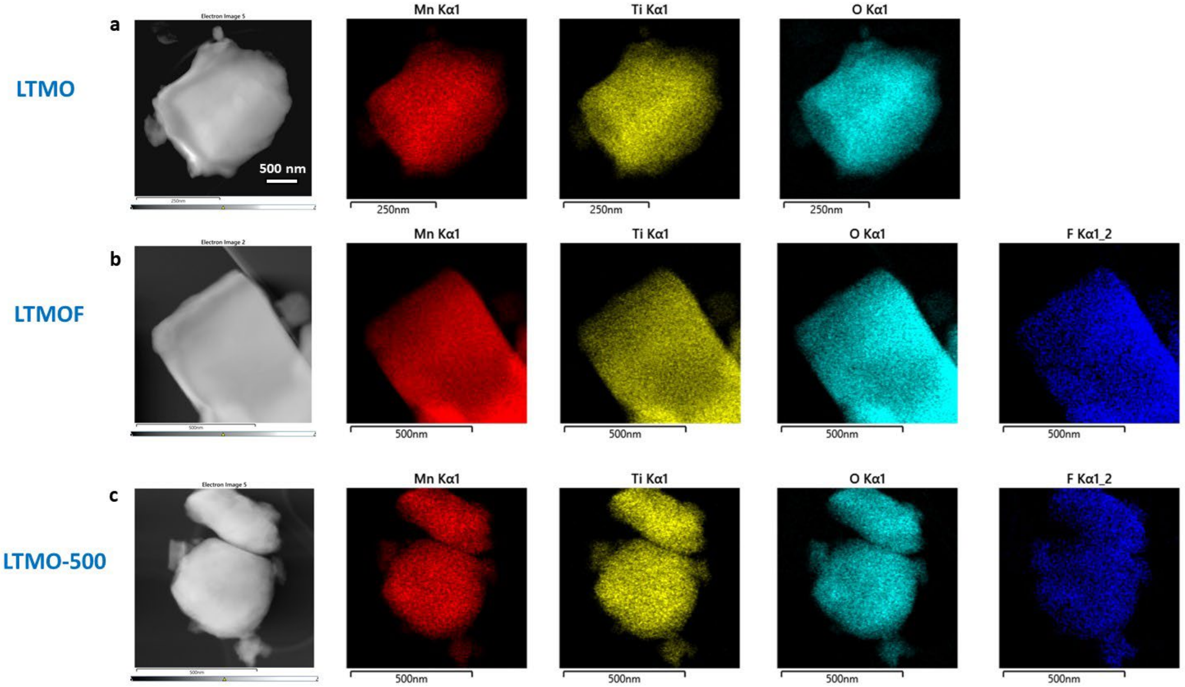

Figure S10. Elemental distribution of the cathode particles. STEM images and corresponding EDS elemental maps of LTMO (a), LTMOF (b), and LTMO-500 (c).

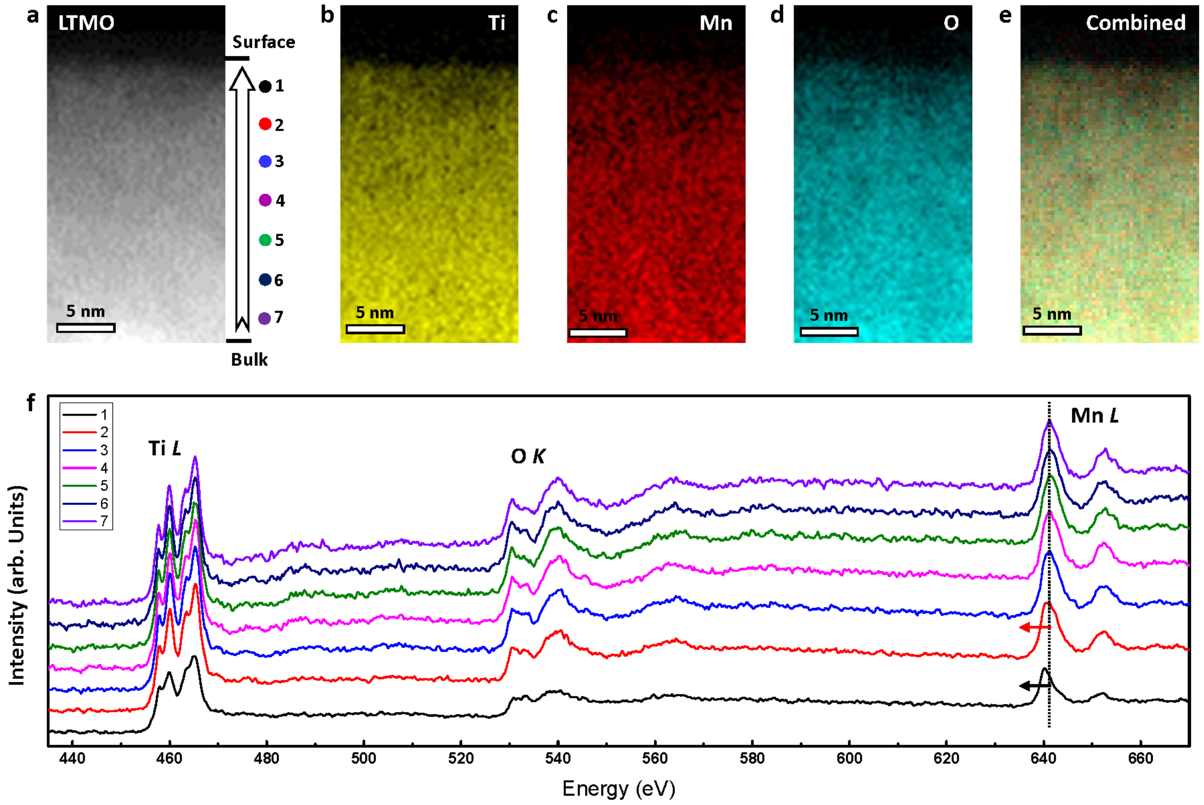

Figure S11. Spatial information of elements in a single particle of LTMO. (a-e) STEM image (a) and corresponding EELS elemental maps (b-e) of LTMO. (f) EELS spectra measured at the 7 different locations marked in (a) (the red and black arrows indicate the chemical shift of the energy loss).

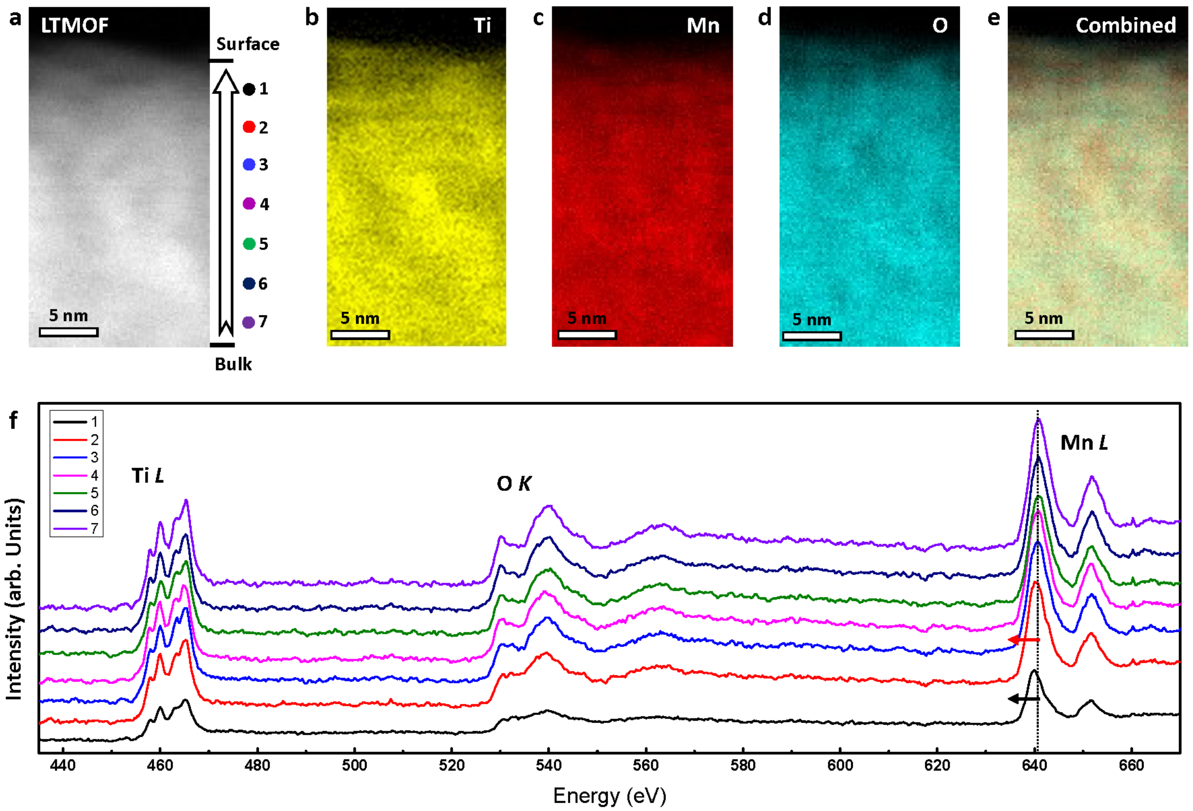

Figure S12. Spatial information of elements in a single particle of LTMOF. (a-e) STEM image (a) and corresponding EELS elemental maps (b-e) of LTMOF. (f) EELS spectra measured at the 7 different locations marked in (a) (the red and black arrows indicate the chemical shift of the energy loss).

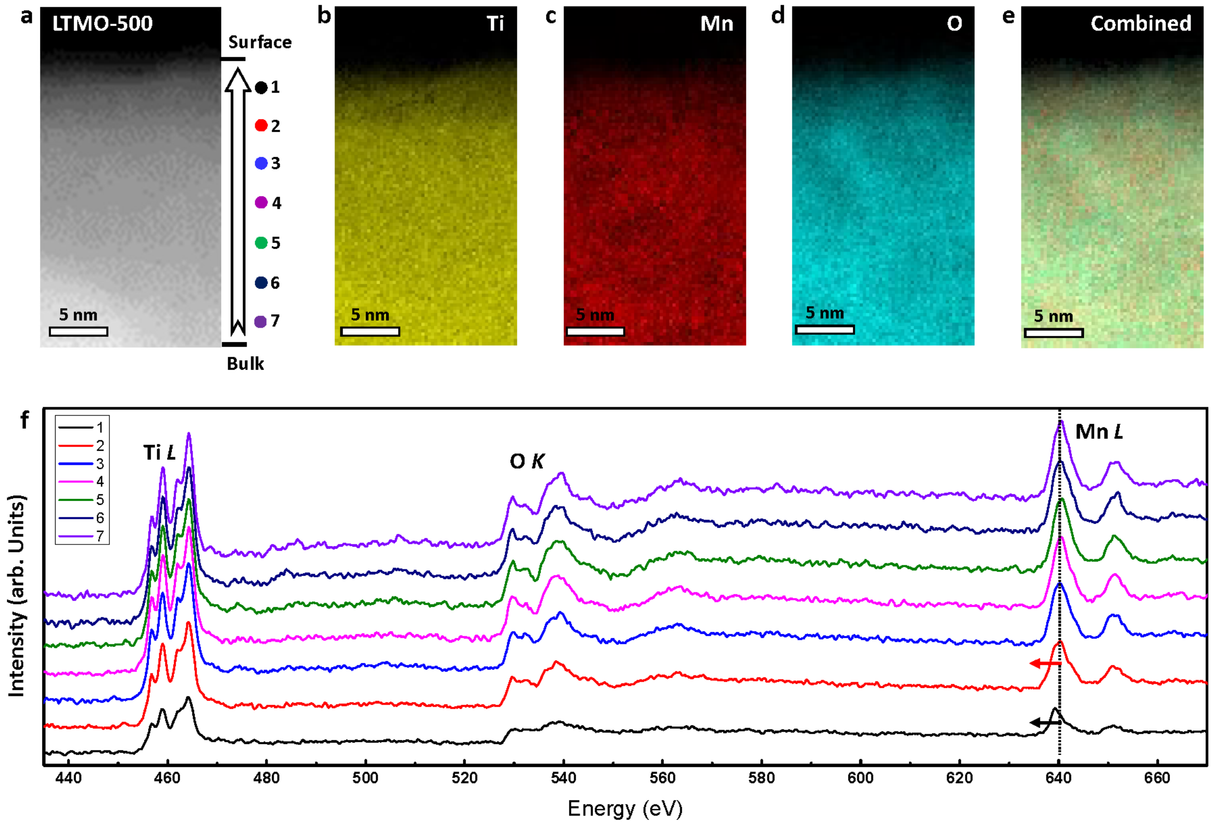

Figure S13. Spatial information of elements in a single particle of LTMO-500. (a-e) STEM image (a) and corresponding EELS elemental maps (b-e) of LTMO-500. (f) EELS spectra measured at the 7 different locations marked in (a) (the red and black arrows indicate the chemical shift of the energy loss).

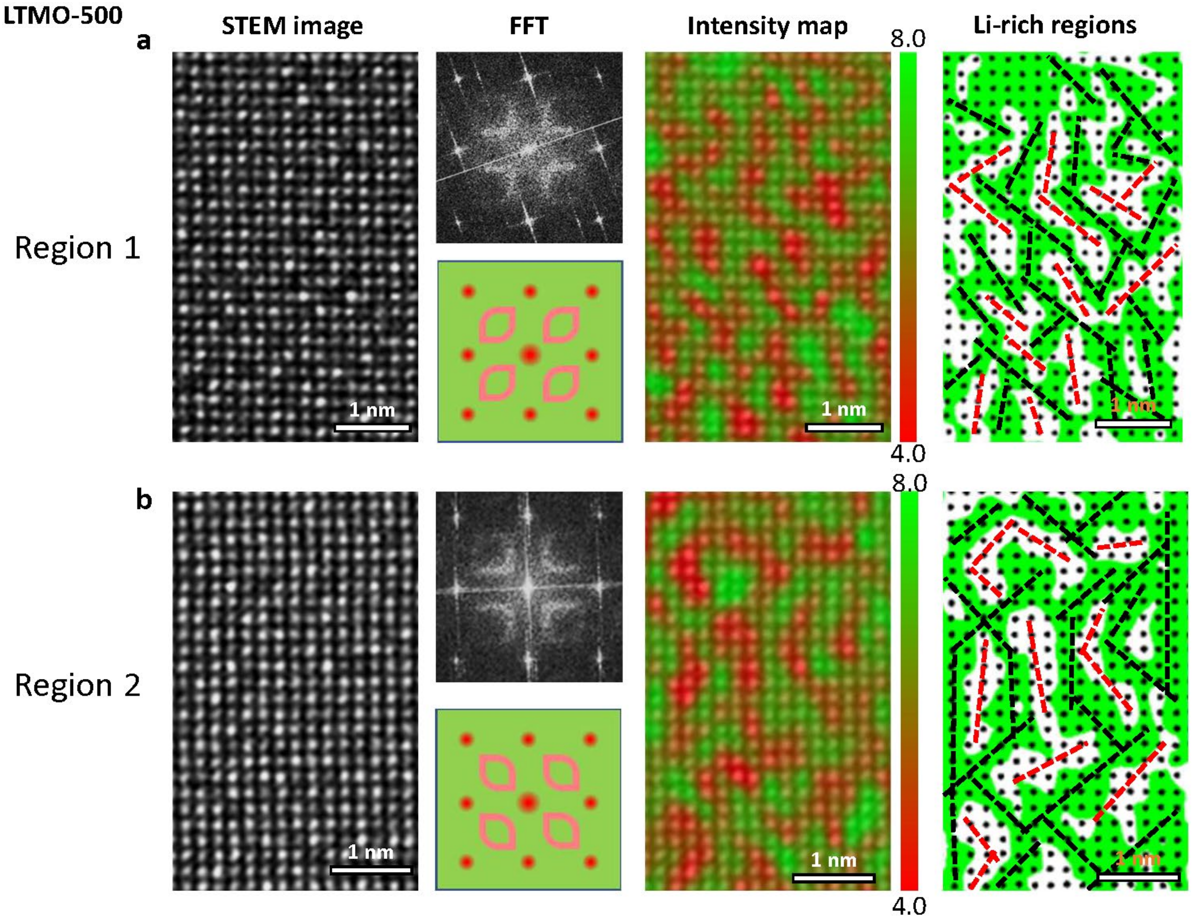

Figure S14. Extraction of Li-rich and TM-rich regions. Atomic-scale STEM *Z*-contrast images (leftmost panels), and the corresponding FFT patterns (second left panels), intensity color maps showing the distribution of both Li-rich (green) and TM-rich (red) nanoregions (third left panels), and the same intensity maps presenting only the Li-rich (green) nanoregions (rightmost panels) for two different local regions in LTMO-500: region 1 (a) and region 2 (b).

Supplementary Tables

Table S1. EDS-measured atomic percentage compositions of LTMO, LTMOF, and LTMO-500.

**Atomic%**	**LTMO**		**LTMOF**		**LTMO-500**	
	Mean	SD	Mean	SD	Mean	SD
**Ti**	14.8	0.8	8.1	0.5	17.0	0.7
**Mn**	15.1	0.8	24.6	0.9	16.0	0.6
**O**	69.3	1.5	64.3	1.2	66.3	1.0
**F**	0.8	0.6	3.0	0.7	0.6	0.5
